# Effectiveness of Specific Techniques in Behavioral Teacher Training for Childhood ADHD Behaviors: Secondary Analyses of a Randomized Controlled Microtrial

**DOI:** 10.1007/s10802-021-00892-z

**Published:** 2022-01-11

**Authors:** Anouck I. Staff, Saskia van der Oord, Jaap Oosterlaan, Rianne Hornstra, Pieter J. Hoekstra, Barbara J. van den Hoofdakker, Marjolein Luman

**Affiliations:** 1 Department of Clinical, Neuro- and Developmental Psychology, Vrije Universiteit Amsterdam, Amsterdam, The Netherlands; 2 Faculty of Psychology and Educational Sciences, KU Leuven, Leuven, Belgium; 3 Department of Pediatrics, Amsterdam Reproduction & Development, Emma Children’s Hospital, Emma Neuroscience Group, Amsterdam UMC, University of Amsterdam, Amsterdam, The Netherlands; 4 Department of Child and Adolescent Psychiatry, University Medical Center Groningen, University of Groningen, Groningen, The Netherlands; 5 Department of Clinical Psychology and Experimental Psychopathology, University of Groningen, Groningen, The Netherlands

**Keywords:** ADHD, Behavioral teacher training, Antecedent-based techniques, Consequent-based techniques, Microtrial

## Abstract

Behavioral teacher training is an effective intervention for children with attention-deficit/hyperactivity disorder (ADHD). Intervention effectiveness may be enhanced by including intervention components that carry the strongest evidence for their effectiveness. A previous article of this group showed that both antecedent- (i.e., stimulus-control) and consequent-based (i.e., contingency management) techniques were highly effective in reducing daily teacher-rated, individually selected problem behaviors in a specific situation of the child. Effects were observed up to three months post intervention. Here, we tested whether effects were also present in teacher-rated and masked DSM-based assessments that comprise the full range of ADHD and oppositional defiant disorder (ODD) symptoms, as well as on teacher-rated impairment. Teachers of 90 children with (subthreshold) ADHD (6–12 years) were randomly assigned to one of three conditions: a short (two sessions), individualized intervention consisting of either a) antecedent-based techniques or b) consequent-based techniques; or c) waitlist. Multilevel analyses showed that both sets of techniques were effective in reducing teacher-rated ADHD symptoms and impairment immediately after the intervention and up to three months later, as compared to waitlist. Masked observations of ADHD behavior were in line with teacher ratings, with effects being most pronounced for inattention. No effects on teacher-rated or masked ODD behavior were found. This study showed that antecedent- and consequent-based techniques were effective in improving classroom ADHD symptoms and impairment. Long-term changes in teacher-rated ADHD are promising. These results extend previous findings and show the potential of short individually tailored interventions in classroom settings as treatment of ADHD symptoms.

**Supplementary Information:**

The online version contains supplementary material available at 10.1007/s10802-021-00892-z.

## Introduction

Behavioral teacher training is an effective intervention to reduce children’s attention-deficit/hyperactivity disorder (ADHD) symptoms and related behavioral problems in the classroom (DuPaul et al., 2012; Evans et al., 2018; Fabiano et al., 2009; Veenman et al., 2016; Ward et al., 2020). Effect sizes of current training programs generally range from small to medium (DuPaul et al., 2012; Ward et al., 2020), thus leaving room for improvement. Insight into which intervention components are effective and which are not may contribute to the development and improvement of behavioral teacher trainings for ADHD (DuPaul et al., 2020; Schatz et al., 2020). However, studies on the effectiveness of separate intervention components are scarce.

Behavioral teacher interventions for ADHD generally include training teachers in the use of both antecedent-based techniques (i.e., stimulus-control techniques such as providing structure and clear instructions) and consequent-based techniques (i.e., contingency management techniques such as praise, reward and planned ignoring) (DuPaul et al., 2022). Teachers are typically taught to combine both sets of techniques, for example by giving a clear instruction to the child to raise his/her hand before speaking and praise the child when doing this (Patterson, 1982). Meta-analytic evidence from behavioral teacher and parent interventions suggests that both antecedent- and consequent-based techniques implemented by teachers and parents are effective to improve children’s ADHD symptoms and oppositional defiant (ODD) behaviors (Gaastra et al., 2016; Leijten et al., 2019). However, meta-analysis only allows testing whether intervention effects are larger for interventions that include a particular intervention component (e.g., training teachers in a set of techniques) as compared to interventions that do not include that particular intervention component (Leijten et al., 2021). Thus each single intervention component is always studied in the context of other intervention components (Lipsey, 2003). Meta-analyses can therefore be used to generate hypotheses about effective intervention components, but whether effect sizes are actually driven by a particular component remains to be studied (Leijten et al., 2021). In contrast, microtrials are experimental designs that can be used to test hypotheses regarding the effectiveness of single intervention components by testing the effects of relatively brief and focused environmental manipulations, such as single intervention components, on proximal outcomes (Howe et al., 2010; Leijten et al., 2015). Such a design allows to study the effectiveness of antecedent- and consequent-based techniques in isolation, which has not been done so far. Therefore, to test the hypotheses about the effectiveness of antecedent- and consequent-based techniques derived from meta-analytic studies (Gaastra et al., 2016; Leijten et al., 2019), our study used a microtrial design to examine the effectiveness of implementing antecedent- and consequent-based techniques in reducing the behavioral problems and impairment children with ADHD often experience in the classroom.

In a previous article of our group (Staff et al., 2021), we analyzed our randomized controlled microtrial using an ecologically momentary assessment (EMA) measure of behavior as outcome measure (Shiffman et al., 2008). Four preselected individual problem behaviors in a specific situation were assessed, and two of these were directly targeted in the intervention. The behaviors and situations thus differed per child-teacher dyad. Examples were ‘difficulties staying focused during individual seatwork’ or ‘talking excessively during whole group teaching’. Following EMA procedures, the four behaviors were daily assessed in the specific situation, maximizing the ecological validity and minimizing recall and retrospection bias that may be observed using traditional questionnaires based on the Diagnostic and Statistical Manual of Mental Disorders (DSM) (Bentley et al., 2019; Shiffman et al., 2008). We showed that antecedent- and consequent-based techniques were equally effective in reducing these four daily teacher-rated problem behaviors in a specific situation. Effects were obtained directly after the intervention (large effects, *d* = 0.89, 0.93, respectively), and remained stable up to three months later.

Nevertheless, two important questions remained unanswered, i.e., whether the promising findings on our EMA outcomes are reflected in: 1) broader assessments of ADHD and ODD behaviors, and 2) impairment. Regarding the first question, we were interested whether effects were also obtained if outcomes comprised the full range of DSM-based teacher-rated ADHD and ODD symptoms assessed on a rating scale, i.e., whether effects could also be observed when teachers were asked to report behaviors averaged over the past week and during all situations, rather than during a specific situation during each day. Using traditional DSM-based questionnaires also provides possibilities to compare results with the findings of other behavioral interventions for ADHD. Further, we were interested whether effects were observed by raters who were not involved in treatment delivery and thus less susceptible to social desirability and/or investment bias (Daley et al., 2014; Sonuga-Barke et al., 2013). Regarding our second question, as functional impairment is often the primary reason for teachers to seek help (Coles et al., 2012), we were interested whether effects are also observed in terms of functional impairment.

The aim of the present study was thus to examine the effectiveness of antecedent- and consequent-based techniques on (1) teacher-rated and masked observations of ADHD and ODD behaviors according to DSM-criteria, and (2) teacher-rated functional impairment. Data were collected in our randomized controlled microtrial that tested two short and individualized behavioral teacher interventions focusing on either antecedent- or consequent-based techniques. Based on our previous findings regarding our EMA outcome, we hypothesized that both sets of techniques would be effective compared to a waitlist control condition in reducing ADHD and ODD symptoms as rated by teachers, both immediately after the intervention as well as at three months follow-up. We expected smaller effect sizes compared to our EMA outcomes (Howe et al., 2010), given that the current measures reflect more distal outcomes. Further, we expected effects to be most pronounced shortly after the intervention compared to three months later (Lee et al., 2012). For our masked assessments of ADHD and ODD behaviors, structured classroom observations were conducted in a (randomly selected) subsample. Classroom observations have shown to be a valid measure to assess ADHD and ODD in the classroom (Minder et al., 2018) and to be sensitive to effects of behavioral interventions (Pelham et al., 2005; Pfiffner et al., 2013). We expected both sets of techniques to be effective in reducing observed ADHD and ODD behaviors (Pfiffner et al., 2013). Finally, we expected both sets of techniques to be effective in reducing functional impairment as rated by the teacher (Groenman et al., 2021).

## Method

### Design

**Fig. 1:**
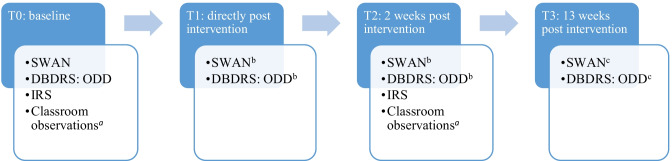
Overview of the outcomes assessed at the different time points. *DBDRS* Disruptive Behavior Disorders Rating Scale, *IRS* Impairment Rating Scale, *ODD* Oppositional Defiant Disorder, *SWAN* Strengths and Weaknesses of ADHD and Normal Behavior. ^a^Classroom observations were conducted in a subset of the sample (*N* = 60). ^b^For analyses on short term effects, outcomes were averaged over T1 and T2. ^c^Longer term effects were assessed in the intervention conditions only

Teachers were randomized to one of two intervention conditions (i.e., antecedent- and consequent-based, see below) or a waitlist control condition. A random list of numbers 1–90 was created to allocate participants to these conditions. Randomization occurred at school level to prevent contamination from teachers receiving different interventions. There was a maximum of two included students per participating teacher. Outcome measures were assessed at three time points: at baseline prior to randomization (T0), during the week immediately after the intervention or the waiting period (T1), and three weeks after the intervention or waiting period (T2). Figure 1 provides an overview of which measures were assessed at each time point. Classroom observations were conducted in a randomly selected subsample of each condition (*n* = 20 per condition). Longer term effects on teacher-rated ADHD and ODD were investigated three months after baseline (T3), in the intervention conditions only. The total study duration was three months (T0-T3) and allowed no holidays between randomization and T2. In case the summer holiday started prior to T3, T3 took place three weeks prior to the end of the school year (but at least four weeks after T2). Because there are no guidelines for reporting on microtrials, we used the CONSORT guidelines for reporting on randomized controlled trials (Moher et al., 2001). More details on the design of the study are available in Staff et al. (2021). This study was registered at the Dutch Trial Register: https://www.trialregister.nl/trial/6616.

### Participants

The study sample comprised 90 regular primary school aged children (grades 1 to 6), from rural and urban areas in The Netherlands, showing ADHD symptoms, and their teachers who were in (self-reported) need of effective management techniques for their student(s). Inclusion criteria were: (a) high levels of ADHD symptoms (> 90th percentile) as rated by teachers on the Inattention and/or Hyperactivity-Impulsivity scale of the Disruptive Behavior Disorders Rating Scale (DBDRS) (Oosterlaan et al., 2008), (b) at least three symptoms (item score ≥ 2) on the Inattention and/or Hyperactivity-Impulsivity scale of the DSM-IV-TR based semi-structured Teacher Telephone Interview (TTI) (Tannock et al., 2002), and (c) a score > 5 (indicating functional impairment, range 0—10) on at least one domain of functioning on a modified version of the teacher-rated Impairment Rating Scale (IRS) (Fabiano et al., 2006). Exclusion criteria were: (a) an estimated full scale IQ < 70, assessed using a short form of the Dutch version of the Wechsler Intelligence Scale for Children-third edition (WISC-III-NL) including the subtests Block Design and Vocabulary (Sattler, 2008), (b) pharmacological treatment for ADHD symptoms during the last month, (c) a diagnosis of autism spectrum disorder or conduct disorder according to the DSM-IV-TR or DSM-5 as reported by parents on a demographic questionnaire, or (d) the teacher being enrolled in a behavioral teacher training aimed at ADHD symptoms or other behavioral problems in the past year.

### Interventions

For the purpose of this microtrial, two short individualized and manualized interventions consisting of two sessions were developed (see Staff et al. (2021) for a detailed description). One intervention included only antecedent-based techniques (referred to as antecedent-based intervention), the other only included consequent-based techniques (referred to as consequent-based intervention). The interventions were based on evidence-based behavioral parent training programs aimed at remediating ADHD symptoms and ADHD related behaviors (Barkley, 1987; McMahon & Forehand, 2003; Van Den Hoofdakker et al., 2007). The first session took place at the school and lasted two hours, the second session was scheduled one week later and took place by video conference, lasting 45 minutes.

At the beginning of the study period, teachers selected four individual problem behaviors per child from a list of 32 ADHD and oppositional behaviors in a specific situation (e.g., difficulties staying focused during individual seatwork) (Staff et al., 2021; Van Den Hoofdakker et al., 2007), from which two behaviors were directly targeted in the intervention. The first session of both interventions consisted of the following steps: (1) providing the teacher with psycho-education on ADHD; (2) selecting the problem behavior, based on the frequency (preferably daily) and severity of behavior; (3) making a behavioral analysis of the behavior by the teacher and therapist; (4) defining desired target behaviors; (5) teaching teachers how to implement either antecedent- or consequent-based techniques (depending on the assigned intervention condition) most optimally, and making a behavioral intervention plan by the teacher and therapist. For each intervention plan, one or more techniques of the assigned condition could be chosen to be part of the intervention plan, based on the behavioral analysis; (6) practicing the intervention plan (i.e., techniques) through visualization or role play; (7) instructing teachers to implement the intervention plan in the classroom for one week, after which the second session took place. The second session started with evaluating the preceding week and adapting the intervention plan, if necessary. Thereafter, steps two to six of the first session were repeated. At the end of the second session, teachers were provided with handouts of the techniques and were instructed to implement both intervention plans directly after the session for at least four weeks. Teachers could contact the therapist if required.

Differences between the two interventions concerned the focus on either antecedent- or consequent-based techniques. More specifically, interventions differed in steps 1, 3, and 5 (see also Table A in Supplementary Information S1). In the antecedent-based intervention teachers were provided with supplemental psycho-education (step 1) on how stimuli evoke behaviors, how executive functioning deficits in children with ADHD may lead to difficulties adapting behavior to stimuli, and how antecedent-based techniques adapt to this by changing the discriminative value of stimuli. The behavioral analysis (step 3) focused on identifying antecedents that elicited the problem behavior. The intervention plan (step 5) in this condition consisted of antecedent-based techniques only (i.e., setting clear rules, providing clear instructions, discussing challenging situations with the child in advance, and providing structure in time and space). These techniques were briefly explained and could be part of the intervention plan. In the consequent-based intervention, teachers’ psycho-education (step 1) was supplemented with specific information on how consequences affect behavior, that children with ADHD may suffer from an altered reward sensitivity that may influence how their behavior is shaped by the environment, and how consequent-based techniques adapt to this by changing the consequences of behavior (Van der Oord & Tripp, 2020). The behavioral analysis (step 3) was targeted at identifying consequences that positively or negatively reinforce the problem as well as desired behavior (i.e., functional behavior assessment, FBA; Dunlap & Kern, 2018). The following consequent-based techniques were explained and integrated in the intervention plan (step 5): praise, reward, planned ignoring, and negative consequences. Shaping was explained and used when the full desired target behavior was not displayed yet. Consequent-based techniques such as token economy and time-out were not included in this intervention given that these also require antecedent-based techniques (e.g., clear rules, structuring by use of individual instructions).

When teachers brought up that they could use techniques from the other intervention (e.g., reward desired behavior in the antecedent-based intervention), the therapists were instructed to explain that the current intervention focused on the trained techniques and therefore the intervention plan consisted of these techniques only. The teacher was advised to implement and/or optimize the trained techniques first and at least until the last week of assessments, to monitor its effectiveness and to decide whether the use of other techniques was needed at a later time. More information on the interventions and examples of intervention plans for every intervention are available in our previous publication (Staff et al., 2021).

#### Therapists and Intervention Fidelity

Interventions were carried out by two psychologists with postgraduate training in behavioral therapy and ADHD (AS and RH) (see also Staff et al. (2021)). Therapists were trained in the program and supervised by licensed supervisors in the postgraduate behavior therapy program with ample experience in behavioral parent and teacher training programs (SvdO and BvdH). Supervision started with individual supervision sessions until quality was sufficient. Thereafter, there were group-based meetings every two weeks with the therapists and supervisors to monitor intervention fidelity (see below) and to provide supervision until the end of the study period. At the beginning of the study period, supervisors checked audiotaped sessions to assess the quality of the session(s) of each condition until optimal quality was reached (maximum scores), as well as to assess intervention fidelity (see below). Quality was rated based on knowledge, structure, and therapeutic process (e.g., providing clear instructions, adequately dealing with resistance), rated on a 3-point Likert scale (1 = *needs work*, 2 = *acceptable*, 3 = *good work*). Both therapists reached optimal quality scores for each condition after the first sessions.

Treatment fidelity was assessed by scoring contamination and by scoring the percentage of addressed session items in each session. The procedure of Abikoff et al. (2013) was used to score contamination. Contamination was defined as a) the therapist recommending the use of non-assigned techniques, b) therapists’ questions or remarks that could elicit teacher’s thoughts or comments on techniques belonging to the non-assigned intervention, or c) the therapist actively supporting and elaborating on the teacher’s suggestion to use of techniques specific to the non-assigned intervention. The contamination score was based on the frequency of contamination occurrences in a session. After optimal quality scores were reached, a random sample of ten percent of the sessions were listened back and scored on intervention fidelity by independent evaluators during the entire study (all intervention sessions were audiotaped). In addition to scoring the selected audiotapes, the percentage of addressed session items was also scored using session-forms that were completed by therapists after each session.

### Outcome Measures

#### Teacher Rating Scales

##### ADHD Symptoms

Teacher ratings of symptoms of inattention and hyperactivity-impulsivity were assessed using the scales Inattention (nine items) and Hyperactivity-Impulsivity (nine items) of the DSM-based Strengths and Weaknesses of ADHD-symptoms and Normal Behavior (SWAN) rating scale (Swanson et al., 2012). Teachers rated a child’s behavior over the past week compared to peers on a 7-point Likert scale (-3 = *far below average* to + 3 = *far above average*). Scores were reverse scored for consistency with other measures used in this study. Scores may range between -27 and 27 for both scales, with higher scores indicating more ADHD symptoms. The internal consistency for the SWAN in this sample was good (*α* = 0.85) and convergent validity has been established (Strengths and Difficulties Questionnaire (SDQ) Hyperactivity scale; *r* = 0.54) (Lakes et al., 2012). The Dutch population based mean scores are *M* = 44.0 (boys)/*M* = 45.7 (girls), *SD* = 8.08 for the Inattention scale and *M* = 43.9 (boys)/*M* = 45.6 (girls), *SD* = 8.63 for the Hyperactivity/Impulsivity scale (scale 1 = *far below average*, 7 = *far above average*) (Polderman et al., 2007).

##### ODD-symptoms

Teacher ratings of symptoms of ODD were measured with the ODD-scale of the DBDRS (Oosterlaan et al., 2008). Teachers rated a child’s behavior over the past week on eight items, using a 4-point Likert scale ranging from 0 (‘*not at all*’) to 3 (‘*very much*’). Scores may range between 0 and 24, with higher scores indicating more ODD symptoms. The internal consistency for the teacher-rated ODD-scale of the Dutch version of the DBDRS is high (*α* = 0.95; *α* = 0.92 in this sample), and convergent validity is strong (IOWA Conners’ Inattention/Overactivity scale; *r* = 0.70, SDQ Hyperactivity scale; *r* = 0.79—0.83) (Oosterlaan et al., 2008).

##### Impairment

An overall measure of functional impairment of the child at school was assessed using an adjusted version of the teacher-rated Impairment Rating Scale (Fabiano et al., 2006). Teachers rated impairment over the past week on the following four areas of functioning: peer, teacher, academics, and classroom. An example of a question is: ‘*How this child’s problems affect his or her relationship with other children?*’. Impairment was rated on a 10-point scale, ranging from 0 (‘*no impairment*’) to 10 (‘*excessive impairment*’), in line with the Dutch system for academic grading. A score above 5 indicated functional impairment on that particular area of functioning. Outcome was the average score on the four items (ranging from 0 to 10).

#### Classroom Observations of ADHD and ODD Behaviors

Classroom observations were conducted in a randomly selected subset of the sample, given the time required for coding (i.e., 570 hours of coding for the subsample analyzed here). For twenty randomly selected children from every condition (67%), classroom observations were coded. The total subsample did not differ from the full sample on baseline characteristics nor in their response to the two interventions studied here as assessed in terms of the proximal outcome (results available from the first author). Observations were conducted when children attended morning lessons in their own classroom led by their primary teacher, and were on similar time and day at both time points (e.g., Tuesday morning at the beginning of the school day) for approximately 90 minutes per child. The first 60 minutes that contained actual lessons were used for coding (e.g., the observation started when children were arriving at the beginning of the day, coding started when the teacher started the first lesson) (coding duration *M* = 57.30 min, *SD* = 9.29 min for T0; *M* = 56.13 min, *SD* = 5.27 min for T2).

A Dutch adapted version of the Ghent University Classroom Coding Inventory (GUCCI; Staff et al., 2020) was used to code behavior, according to four scales: Attention Problems (i.e., visual attention to task), Motor Hyperactivity (i.e., motor movements), Verbal Hyperactivity (i.e., talking or other vocalizations), and Oppositional Behavior (i.e., arguing, anger). Each scale comprised a categorical variable of behavior to be coded as absent or present, categories within each scale were mutually exclusive (e.g., Motor Hyperactivity consisted of the levels no motor hyperactivity and motor hyperactivity, see Table 1). Scales were coded using continuous sampling, indicating that all behaviors were coded throughout the coding period. For the Attention Problems scale, the percentage of time off-task was calculated by dividing the total time off-task by the total time coded in which the child was expected to be involved in class activities (sum of the time of on- and off-task). When no involvement in class activities was expected, the interval was coded as no-task. For the behavioral categories Motor Hyperactivity and Verbal Hyperactivity, percentage of total time the behaviors motor hyperactivity and verbal hyperactivity occurred was calculated. For Oppositional Behavior, frequency of oppositional behavior served as outcome (Staff et al., 2020).

**Table 1:** Operational definitions of observed behaviors using the GUCCI

Scale	Coding category	Description	Outcome variable in statistical analysis
Attention Problems	On-task	The child is involved in activities that are expected by the teacher (e.g., paying visual attention to task or to the teacher), and is following the teacher’s instructions and requests	
	Off-task	The child is involved in activities that are not expected by the teacher for at least two seconds (e.g., not working on assignments, daydreaming)	% of time
Motor Hyperactivity	No motor hyperactivity	The child has no difficulty sitting down. Little movements of arms, hands, feet, or legs are accepted and no gross movements that are observably annoying or disturbing peers are shown	
	Motor hyperactivity	The child is not sitting still on his/her chair (e.g., overturns or swings his/her chair, squirms in chair). The child shows small movements that are annoying or disturbing for peers (e.g., tapping with a pen). The child is not sitting on the chair (e.g., standing up without permission, sitting on their knees) or is walking or running through the classroom	% of time
Verbal Hyperactivity	No verbal hyperactivity	The child is quiet, or the child talks in reaction to the teacher’s request	
	Verbal hyperactivity	The child is talking or making vocal sounds (e.g., whispering to self, humming)	% of time
Oppositional behavior	No oppositional behavior	The child does not show any oppositional behavior, anger, aggression, or antisocial behavior against others	
	Oppositional behavior	The child shows oppositional behavior against the teacher (e.g., refuses something). The child shows angry behavior (e.g., shows tantrum)	Frequency

*GUCCI* Ghent University Classroom Coding Inventory

Observations were coded by fourteen graduate psychology students (i.e., observers), who were individually trained by the first author in at least two sessions of two hours. Observers coded a maximum of two scales, in order to increase accuracy and inter-observer reliability (i.e., four observers coded Attention Problems, five others coded Motor Hyperactivity, and five others coded Verbal Hyperactivity and Oppositional Behavior). Observers were masked to treatment condition of the child as well as to whether an observation was conducted at pre- (T0) or post-intervention (T2). During the training they were introduced to the behavioral categories of the scale(s) and the coding system. Observers practiced coding until inter-observer agreement with the trainer reached ≥ 0.80 (see for detailed information: Staff et al. (2020)). Given that we used continuous coding, rather than time sampling, inter-observer agreement was based on the percentage of time behaviors were scored in the same category by both raters and ranged between 82.9% and 99.8%. Additionally, intra-class coefficients (ICC, based on a one-way random model, Hallgren (2012)) for each scale were calculated to have an estimation of inter-rater reliability corrected for measurement error. Inter-rater agreement was excellent (*ICC* ≥ 0.86) for this sample. Convergent validity of the GUCCI was adequate (*r* = -0.04—0.29), although relatively low correlations between rating scale scores and observational scores indicate that both instruments measure different aspects of ADHD and ODD behavior (see for a detailed evaluation: Staff et al. (2020)).

### Procedure

This study was carried out between April 2017 and April 2019. Teachers were recruited through school principals, school collaboration networks, and an outpatient mental health clinic. Teachers showing interest in participation in the study received an information letter explaining the research aims and responsibilities of all parties involved. Teachers who agreed in participating enlisted one to two children showing profound and impairing ADHD symptoms in the classroom, and informed parents about the study (i.e., provided them with the information letter and informed consent). Written consent was obtained from teachers, parents, and children older than 11 years. After receiving consent, teachers administered the ADHD scales of the DBDRS and TTI to screen for eligibility. If inclusion criteria were met, baseline assessments (T0) took place through teacher rating scales and classroom observations, all conducted in the same week. For the classroom observations, observers were introduced as interns. Teachers explained to children that the interns had to observe how children are working during lessons in different classes for study purposes. To prevent target children being aware of being subject of the observations, cameras were positioned in a corner at the front of the classroom, targeted at the whole classroom (but zoomed in at a particular child). Randomization occurred after baseline assessments were completed. Teachers of children in the waitlist condition were allowed to receive care as usual during the study period, and were offered the possibility to use a self-directed behavioral teacher program targeting ADHD symptoms immediately after T2 (PR Program, Veenman et al., 2016). Longer term effects at T3 were therefore only explored in children of teachers in the active intervention arms and were only assessed by teacher ratings. The local medical ethical committee waived the need for medical ethical approval (University Medical Center Groningen, 2016/198).

### Statistical Analysis

Analysis of variance (ANOVA), and chi-squared or Fisher’s exact tests were used to compare groups on the demographic variables assessed at baseline.

Data were analyzed on an intention-to-treat basis. To compare the intervention conditions to the waitlist condition and to each other, multilevel analyses (mixed model) were conducted in Stata (version 16). Missing data was random (≤ 5%) for all outcomes, and was taken into account in multilevel analysis (Twisk et al., 2013). Four hierarchical levels were distinguished: observations (level 1), nested within children (level 2), nested in classrooms (level 3), and nested in schools (level 4). Random intercepts at classroom and school level were only included if significantly improving model fit as determined by Likelihood Ratio Test. We inserted condition (waitlist, antecedent, consequent) as between subjects’ factor, and time (T1, T2) as within variable. Baseline scores (T0) of the outcome were inserted as fixed factor, in order to control for problems at baseline. We investigated short-term effects of condition (averaged over T1 and T2) to compare the intervention conditions to the waitlist condition, and to compare the two intervention conditions to each other. Because effects were similar for T1 and T2 on the proximal outcome (see Staff et al., 2021), we used an aggregated outcome measure for the current study. Longer term effects were assessed by examining whether problem behaviors remained stable from T2 to T3 within each intervention condition (i.e., whether the development of problem behaviors from T2 to T3 changed significantly). Two measures of hyperactivity were included in the classroom observations (i.e., motor and verbal), therefore alpha level was set at 0.05/2 for these outcomes. Given the lower number of participants for the observational measure in each separate condition, we explored whether weaker short-term effects on the classroom observations may have remained undetected using sensitivity analyses. Therefore, we combined the antecedent and consequent condition into one “active” intervention condition (*n* = 40) and compared this to the waitlist condition. Effect sizes (Cohen’s *d*) were calculated by dividing the difference in mean scores between two conditions averaged over T1 and T2 by the pooled SD (Rosnow & Rosenthal, 1996), with 0.20, 0.50, and 0.80 as thresholds for small, medium, and large effects, respectively.

To examine intervention fidelity (Abikoff et al., 2013), we compared the intervention conditions on the contamination scores and the average percentage of addressed session items (as rated by therapists and independent coders) by using independent *t-*tests. We also asked teachers in the antecedent and consequent condition to rate whether they would recommend the intervention to colleagues (yes, no, neutral) at T3 as an indication of the feasibility of the interventions.

## Results

Thirty children (from 25 teachers of 17 schools) were allocated to the antecedent condition, 30 children (from 26 teachers of 18 schools) to the consequent condition, and 30 children (from 26 teachers of 17 schools) to the waitlist condition. Table 2 displays demographic characteristics of the sample. Children randomized to the three conditions did not differ on any of the screening characteristics (*p* > 0.132), with the exception of hyperactivity-impulsivity symptoms on the TTI and DBDRS on which lower ratings were obtained for children in the antecedent condition than for children in the consequent condition (TTI) and waitlist condition (TTI and DBDRS). Parents reported that 23 children (26%, evenly distributed over conditions, see Table 2) had been clinically diagnosed with ADHD and none had been diagnosed with ODD. Based on the TTI, 42 children (47%) met the criteria for DSM-V ADHD (i.e., at least six out of nine symptoms in at least one domain) and 10 children (11%) met the criteria for DSM-V ODD within the school setting.

**Table 2:** Sample description and baseline comparisons

	AC (*n* = 30)	CC (*n* = 30)	WC (*n* = 30)	Group comparisons
Age at assessment in years	8.53 (1.63)	9.08 (1.63)	8.76 (1.52)	*F*(2, 89) = 0.88, *p* = 0.420
Sex, *n* (*%*) boys	23 (77)	23 (77)	28 (93)	*χ*^2^ = 3.81, *p* = 0.150
IQ	99.77 (11.04)	99.33 (14.28)	104.07 (10.05)	*F*(2, 89) = 1.45, *p* = 0.241
SES^a^	5.22 (1.24)	5.24 (1.12)	5.00 (1.03)	*F*(2, 88) = 0.41, *p* = 0.664
Caucasian, *n* (*%*)	28 (93)	27 (90)	30 (100)	*Fisher’s exact* = 0.294, *p* = 0.363
ADHD diagnosis, *n* (*%*)	8 (27)	8 (27)	7 (23)	*χ*^2^ = 0.12, *p* = 0.943
Other psychiatric diagnosis, *n* (*%*)	0 (0)	3 (10)	0 (0)	*Fisher’s exact* = 4.22, *p* = 0.104
TTI symptom severity				
Inattention	4.30 (1.58)	5.00 (1.86)	4.13 (1.91)	*F*(2, 89) = 1.99, *p* = 0.143
Hyperactivity-Impulsivity	2.97 (1.85)	4.83 (2.38)	4.60 (2.22)	*F*(2, 89) = 6.65, *p* = 0.002 (CC, WC > AC)
ODD	1.10 (1.45)	1.07 (1.46)	1.23 (1.61)	*F*(2, 89) = 0.10, *p* = 0.903
CD	0.00 (0.00)	0.00 (0.00)	0.13 (0.51)	*F*(2, 89) = 2.07, *p* = 0.132
DBDRS				
Inattention	16.90 (4.96)	17.50 (3.92)	16.00 (5.57)	*F*(2, 89) = 0.72, *p* = 0.488
Hyperactivity-Impulsivity	13.17 (6.21)	15.73 (6.49)	17.57 (6.60)	*F*(2, 89) = 3.54, *p* = 0.033 (WC > AC)
IRS impairment^b^				
Number of domains	3.07 (0.98)	2.97 (1.27)	3.24 (0.88)	*F*(2, 84) = 0.45, *p* = 0.638
Average score	6.22 (1.65)	6.14 (1.97)	6.29 (1.28)	*F*(2, 84) = 0.52, *p* = 0.948
*Teacher ratings*				
SWAN				
Inattention	15.03 (4.41)	14.17 (5.11)	15.07 (5.07)	*F*(2, 89) = 0.33, *p* = 0.721
Hyperactivity-Impulsivity	13.57 (6.77)	13.77 (6.35)	16.83 (6.26)	*F*(2, 89) = 2.41, *p* = 0.096
DBDRS				
ODD	8.00 (6.45)	5.00 (5.09)	8.97 (5.32)	*F*(2, 89) = 4.02, *p* = 0.021 (AC, WC > CC)
*Parent ratings*^*c*^				
SWAN				
Inattention	5.31 (8.58)	9.21 (7.54)	5.86 (5.53)	*F*(2, 83) = 2.37, *p* = 0.100
Hyperactivity-Impulsivity	6.08 (8.24)	9.41 (6.20)	9.83 (6.61)	*F*(2, 83) = 2.31, *p* = 0.106
DBDRS				
ODD	5.90 (4.94)	5.28 (3.43)	6.17 (4.40)	*F*(2, 88) = 0.33, *p* = 0.719
*Classroom observations*^*d*^				
Inattention *%*	27.23 (15.96)	28.97 (10.88)	30.56 (16.66)	*F*(2, 59) = 0.26, *p* = 0.774
Motor hyperactivity *%*	30.37 (19.63)	40.35 (20.47)	32.60 (15.67)	*F*(2, 59) = 1.57, *p* = 0.217
Verbal hyperactivity *%*	5.73 (4.87)	9.08 (7.83)	10.69 (6.75)	*F*(2, 59) = 2.94, *p* = 0.061
Oppositional behavior *K*	0.30 (1.13)	0.45 (1.00)	1.65 (3.08)	*F*(2, 59) = 2.79, *p* = 0.070

*M* and *SD* are depicted unless otherwise stated

*AC* antecedent condition, *ADHD* attention-deficit/hyperactivity disorder, *CC* consequent condition, *CD* conduct disorder, *DBDRS* Disruptive Behavior Disorder Rating Scale, *IRS* Impairment Rating Scale, *K *count, *ODD* oppositional defiant disorder, *SES* socioeconomic status, *SWAN* Strengths and Weaknesses of ADHD and Normal Behavior, *TTI* Teacher Telephone Interview, *WC* waitlist control condition

^a^SES was measured by parental educational level (average of both parents) through the Dutch classification system (1 = no education completed, 2 = early childhood education, 3 = primary education, 4 = lower secondary education, 5 = upper secondary education, 6 = undergraduate school, 7 = graduate school, 8 = post-graduate education) (CBS, 2016)

^b^Five children started directly after the summer holiday, but were screened before the summer holiday. As teachers were not able to rate impairment in the first week of school, for these children functional impairment ratings were missing

^c^Missing parent ratings: 1 parent (CC) did not fill in any questionnaire, and 5 other parents (4 AC, 1 CC) did not fill in the SWAN

^d^For analyses on classroom observations a subsample (*n* = 60) of children was used, see Supplementary Information S2 (Table B) for a description of this subsample

Characteristics of the subset of the sample for which the classroom observations were coded is described in Supplementary Information S2 (Table B).

### Effects of Techniques

Intervention effects on all short-term outcomes are depicted in Table 3 (means and standard deviations at all four time points on all outcomes are reported in Supplementary Information S3, Table C, and Figures of the development of behavior over time for all outcomes are reported in Supplementary Information S4, Fig. A). For all outcomes, the levels ‘school’ and ‘classroom’ did not affect intercept variance. Hence these levels were removed from the models that now included two levels (observations clustered in students). Only for the Verbal Hyperactivity scale of the GUCCI the level classroom improved model fit and was thus included in the model. Two teachers discontinued participation after T0 (change of job and illness, *n* = 1 for the antecedent and waitlist condition), and two other teachers (*n* = 1 for the consequent and waitlist condition) discontinued after T2 due to personal problems.

**Table 3:** Short term effects of the antecedent- and consequent-based techniques on all outcomes

	AC vs WC			CC vs WC			AC vs CC		
**Teacher ratings**	*B (SE)*	*p*	*d (95% CI)*	*B (SE)*	*p*	*d (95% CI)*	*B (SE)*	*p*	*d (95% CI)*
Inattention symptoms (SWAN)	-3.41 (1.13)	0.003	0.57 (0.30–0.84)	-2.04 (1.13)	0.071	0.34 (0.08–0.60)	-1.37 (1.12)	0.223	0.23 (-0.03–0.49)
Hyperactivity-impulsivity symptoms (SWAN)	-4.70 (1.19)	<0.001	0.69 0(0.42–0.96)	-3.05 (1.19)	0.010	0.45 (0.19–0.72)	-1.65 (1.16)	0.155	0.24 (-0.02–0.50)
ODD-symptoms (DBDRS)	-1.26 (0.97)	0.194	0.23 (-0.03–0.49)	-0.39 (1.01)	0.699	0.07 (-0.19–0.33)	-0.87 (0.99)	0.378	0.16 (-0.10–0.42)
Impairment (IRS) average score	-1.08 (0.48)	0.023	0.62 (0.35–0.89)	-1.11 (0.48)	0.021	0.63 (0.36–0.90)	0.03 (0.44)	0.954	0.01 (-0.25–0.27)
**Classroom observations**^*a*^									
Inattention (*%*)^*b*^	-8.82 (4.33)	0.042	0.55 (0.28–0.82)	-10.48 (4.26)	0.014	0.65 (0.38–0.92)	1.66 (4.38)	0.704	0.10 (-0.16–0.36)
Motor hyperactivity (*%*)^*b*^	-6.06 (4.90)	0.216	0.34 (0.08–0.60)	-7.99 (4.91)	0.103	0.45 (0.19–0.72)	1.94 (5.03)	0.700	0.11 (-0.15–0.37)
Verbal hyperactivity (*%*)^*c*^	3.10 (2.48)	0.212	0.42 (0.16–0.69)	-3.49 (2.45)	0.154	0.47 (0.20–0.74)	6.59 (2.53)	0.009	0.88 (0.61–1.15)
Oppositional behavior (*K*)^*b*^	-0.90 (0.54)	0.097	0.43 (0.17–0.70)	-0.58 (0.54)	0.278	0.28 (0.02–0.54)	-0.32 (0.52)	0.544	0.15 (-0.11–0.41)

The fixed effect of group represent group differences averaged over T1 and T2 while controlling for baseline scores (T0)

The control condition or the consequent condition was used as reference group

*AC* antecedent condition, *ADHD* attention-deficit/hyperactivity disorder, *CC* consequent condition, *DBDRS* Disruptive Behavior Disorder Rating Scale, *ODD* oppositional defiant disorder, *IRS* Impairment Rating Scale, *K *count, *SWAN* Strengths and Weaknesses of ADHD and Normal behavior rating scale, *WC* waitlist-control condition

^a^Classroom observations were conducted in a subsample of children (*n* = 60), at T0 and T2. For descriptions of this sample see Supplementary Information S2 (Table B)

^b^Level child was included in the model

^c^Levels child and class were included in the model

#### Teacher-rated ADHD Symptoms

Results showed that for the teacher-rated inattention scale (effects averaged over T1 and T2 while controlling for T0, see Table 3) there was a medium sized, significant reduction of symptoms for the antecedent condition as compared to the waitlist condition, and a non-significant (although trend), small to medium effect for the consequent condition compared to the waitlist condition. Regarding teacher-rated hyperactivity-impulsivity symptoms, both intervention conditions showed a significant decrease in symptoms as compared to the waitlist condition, with medium to large effects. Both intervention conditions did not significantly differ from each other on the two symptom domains.

Analyses of longer-term changes as assessed with teacher ratings revealed that inattention symptoms remained low (even decreased) from T2 to T3 in both intervention conditions (for antecedent: *B* = -4.19, *SE* = 0.90, *p* < 0.001; for consequent: *B* = -2.21, *SE* = 0.88, *p* = 0.012). Approximately similar effects were found for hyperactivity-impulsivity symptoms (for antecedent: *B* = -2.57, *SE* = 0.99, *p* = 0.001; for consequent: *B* = -2.38, *SE* = 0.97, *p* = 0.015).

#### Observed ADHD Symptoms

Masked assessments of ADHD behavior using classroom observations revealed that there was a decrease in inattention in children in both the antecedent- and consequent condition as compared to children in the waitlist condition from T0 to T2 with medium to large short-term effects, see Table 3. Post-hoc analyses showed that this is likely to be explained by a trend significant increase in inattention in the waitlist condition over time (*B* = 5.77, *SE* = 3.36, *p* = 0.086), while the decrease in attention problems within the antecedent- and consequent conditions was non-significant (*B* = -1.41, *SE* = 3.49, *p* = 0.687; *B* = -3.91, *SE* = 3.42, *p* = 0.254, respectively). For motor hyperactivity and verbal hyperactivity, no significant reductions were observed when comparing the intervention conditions to the waitlist condition. There were no significant differences between the antecedent and consequent condition in the effectivity of the two interventions on observed attention problems and motor hyperactivity. For verbal hyperactivity, however, results showed that verbal hyperactivity increased over time in the antecedent condition as compared to the consequent condition with a medium to large effect. Post-hoc analyses within each condition revealed that there was a significant increase in verbal hyperactivity in the antecedent condition from T0 to T2 (*B* = 5.24, *SE* = 2.05, *p* = 0.010), while verbal hyperactivity remained stable from T0 to T2 in the consequent condition (*B* = -2.91, *SE* = 2.02, *p* = 0.149).

#### Teacher-rated ODD Symptoms

Analyses of short-term effects showed that there were no significant reductions in teacher-rated ODD symptoms (DBDRS) in the intervention conditions compared to the waitlist condition, and when comparing both intervention conditions to each other, see Table 3.

Analyses of longer-term effects (T2 to T3) of teacher-rated ODD symptoms showed that there were no significant changes in ODD symptoms in any of the intervention conditions (for antecedent: *B* = -0.46, *SE* = 0.67, *p* = 0.492; for consequent: *B* = -0.96, *SE* = 0.67, *p* = 0.153).

#### Observed ODD Symptoms

No significant reductions in ODD symptoms in the intervention conditions compared to the waitlist condition were obtained with the masked classroom observations, see Table 3.

#### Impairment

Significant and similar reductions of teacher-rated functional impairment were found in both intervention conditions as compared to the waitlist condition from T0 to T2, see Table 3, with medium effect sizes.

#### Sensitivity Analyses for Classroom Observations

Results showed a medium sized decrease in attention problems from T0 to T2 in the “active” intervention group as compared to the waitlist group (*B* = -9.68, *SE* = 3.70, *p* = 0.009, *d* = 0.60). There was also a small to medium sized decrease (trend significant) in motor hyperactivity obtained between the “active” intervention condition compared to the waitlist group (*B* = -7.02, *SE* = 4.22, *p* = 0.096, *d* = 0.40). No significant differences in verbal hyperactivity and oppositional behavior were observed between the “active” intervention and waitlist condition (*B* = -0.29, *SE* = 2.23, *p* = 0.897, *d* = 0.04; *B* = -0.74, *SE* = 0.48, *p* = 0.120, *d* = 0.35, respectively).

#### Intervention Fidelity and Feasibility

Contamination occurred once in one session of the consequent condition and did not occur in any of the sessions of the antecedent condition. Contamination scores did not differ between the two interventions: *t*(3.00) = -1.00, *p* = 0.391. The average percentage of addressed session items was high in the antecedent and consequent condition according to both therapists’ self-report (98.9% and 99.4% respectively) and recorded sessions (98.0% and 97.8% respectively). Most teachers would recommend the training to colleagues (antecedent: *n* = 21 [88%]; consequent; *n* = 17 [77%]), with no differences between the two conditions (*χ*^*2*^ = 0.84, *p* = 0.361).

## Discussion

Using a microtrial design, this study was aimed to gain insight into whether previously found effects of antecedent- and consequent-based techniques in teacher training for children with ADHD on EMA outcomes (Staff et al., 2021), were also reflected in broader assessments. More specifically, we examined the effectiveness of both sets of techniques on teacher ratings that comprise the full range of DSM-criteria for ADHD and ODD behaviors, masked classroom observations of ADHD and ODD behaviors, as well as teacher-rated functional impairment.

Effects on DSM-based teacher-rated ADHD were mostly in line with our previously reported findings (Staff et al., 2021), and with the broader literature on teacher trainings for ADHD (DuPaul et al., 2012; Evans et al., 2018; Fabiano et al., 2009; Ward et al., 2020). The previous article showed that both interventions were effective in reducing four daily-rated, individually selected, problem behaviors of the child in specific situations, of which two were directly targeted in the intervention. The current article extends these findings by showing that intervention effects were also present in reductions ADHD symptoms according to DSM-criteria as rated by teachers averaged over the past week and all situations, and in reductions of teacher-rated impairment. Teacher-rated ADHD symptoms in the antecedent and consequent conditions even improved up to levels close to the population based mean (Polderman et al., 2007), while children in the waitlist condition continued to score one standard deviation above the population mean. As effects were obtained on multiple measures (more and less susceptible to bias) and outcomes, this strongly confirms the effectiveness of the interventions.

No significant differences were observed in the effect sizes of both interventions compared to the waitlist condition. This is in contrast to meta-analytic results showing that effect sizes of teacher programs that include consequent-based interventions are somewhat larger than programs that include antecedent-based interventions (Gaastra et al., 2016). However, as these meta-analytic findings only present evidence in the context of other intervention components (Lipsey, 2003), our findings support the importance of testing hypotheses using experimental (microtrial) designs in order to draw more firm conclusions on the effectiveness of intervention components (Leijten et al., 2021). Another explanation for the finding that our antecedent- and consequent-based interventions were both effective compared to waitlist condition with similar effect sizes, may be that antecedent-based interventions included in the meta-analysis by Gaastra et al. (2016) were mostly general educational accommodations (e.g., extended time) of which the evidence base is limited (Lovett & Nelson, 2020). Furthermore, most of these antecedent-based interventions were not tailored to individual needs of the child, while included consequent-based interventions were. In the current study, both interventions were tailored to individual needs (using the behavioral analysis), which may have increased the relative effectiveness of antecedent-based interventions as compared to consequent-based interventions (Dunlap & Kern, 2018; Harrison et al., 2019). When comparing the intervention conditions to the waitlist condition, there were even indications that the antecedent-based intervention was somewhat more effective than the consequent-based intervention in reducing teacher-rated inattention symptoms (i.e., medium-sized effect for antecedent-based intervention versus a small to medium-sized effect for consequent-based intervention). A similar study of our group into the effectiveness of both types of intervention components in behavioral parent training for ADHD also found this pattern (Hornstra et al., 2021). As argued by Hornstra and colleagues, it may be that antecedent-based techniques potentially require less time and effort of teachers to implement during the training as compared to consequent-based techniques, because antecedent-based techniques focus on the prevention of problem behavior and can be implemented regardless of child behavior. In addition, before consequent-based techniques can be effective, children may have to be repeatedly exposed to alternated contingencies in order to adapt their behavior, while antecedent-based techniques may have direct effects (Owen et al., 2012).

Our findings were in line with studies showing that teacher training has longer term effects over three months (DuPaul et al., 2012; Evans et al., 2018; Fabiano et al., 2009; Ward et al., 2020). Contrary to our expectations, there were even indications for the three months follow-up that teacher-rated ADHD symptoms further improved, regardless of the techniques used, while such effects were not observed for our proximal outcome (i.e., these effects remained stable from post-intervention to follow-up; Staff et al. (2021)), and behavior often deteriorates after treatment is withdrawn (Lee et al., 2012). However, we did not include the waitlist condition at T3 as teachers in this condition were offered treatment after T2, so our results need to be confirmed in future studies.

Further, our findings on masked observations of inattention were consistent with effects obtained with teacher ratings, suggesting that effects on inattention were not affected by possible social desirability and/or investment bias (Daley et al., 2014; Sonuga-Barke et al., 2013). Compared to the waitlist condition, observed attention problems decreased in the active conditions, confirming the positive (and protective) effects of the interventions. Intervention effects on masked hyperactivity-impulsivity were in the same direction as teacher ratings although effects did not reach statistical significance. This is likely to be explained by the limited number of subjects included in our masked analyses, reducing power. Observed verbal hyperactivity, however, did not show such a pattern, and even increased in the antecedent condition over time. Although we cannot fully explain this finding, this may be related to the low baseline levels of this behavior in the antecedent condition (5.7%, see Table 2) compared to the other conditions, while at T2, group differences in verbal hyperactivity between conditions were small. Further research in larger samples is needed to conclude on the effectiveness of the sets of techniques on masked outcomes of hyperactivity.

In contrast to ODD behaviors as measured with the daily EMA ratings (Staff et al., 2021) and meta-analytic results showing effects of behavioral interventions on ODD symptoms (Daley et al., 2014; Leijten et al., 2019), we did not observe effects of the specific techniques on teacher-rated ODD symptoms, neither on the short term, nor on the longer term, nor on classroom observations of oppositional defiant behaviors. This may be explained by the current sample in which children had low levels of baseline ODD symptoms, possibly indicating that there was not enough room for improvement on ODD behavior. However, given that we obtained large effects on the daily ratings of oppositional behavior assessed with the proximal EMA measure, one may also argue that a proximal measure such as daily ratings using EMA may be more sensitive to observe effects compared to measures assessing broadly defined ODD behavior.

Although the results of our study are promising, there are limitations to note. First, this study was powered on our primary outcome and therefore power for the secondary outcomes reported here may have been too low (Jakobsen et al., 2019), possibly leading to small effects being undetected. This seems particularly relevant for antecedent versus consequent comparisons as these are both active conditions. Second, classroom observations were conducted only in a subset of the sample, given the time-intensive nature of coding of the observations, and may have led to undetected small effects. However, the effects obtained for attention problems were robust and provide important corroborative information next to our proximal daily ratings and questionnaire ratings for the effectiveness of both sets of techniques. A third limitation is that we have not quantified teacher implementation of the techniques in the classroom (neither quality or dose), and such it cannot be used as a moderator in the analyses. Fourth, our sample predominantly included children with subthreshold ADHD symptoms and low levels of ODD symptoms. Although our results provide useful information for children with (subthreshold) ADHD, effects may not be generalizable to children with more severe ADHD and/or ODD symptoms. Further, our sample was nearly 100% Caucasian and we lack insight into other relevant child (e.g., parental income) and teacher (e.g., race) factors, which may limit the representativeness or our sample.

### Conclusions and Clinical Implications

This randomized controlled microtrial showed that antecedent- and consequent-based techniques are effective in reducing children’s ADHD symptoms in the classroom, as assessed by teacher-rated DSM-based measures of ADHD symptoms and functional impairment, as well as masked observations of inattention. These findings extend our previously obtained results on a proximal EMA outcome (Staff et al., 2021).

Importantly, the effect sizes of these brief and individualized interventions on our secondary outcomes appear similar to those of full and longer interventions often containing both sets of techniques (DuPaul et al., 2012; Evans et al., 2018; Fabiano et al., 2009; Ward et al., 2020). As described previously (Staff et al., 2021), the current interventions were short and individualized and were based on functional behavioral analysis of the child’s problem behavior (FBA; Dunlap & Kern, 2018), which may have added to their effectiveness (Chronis et al., 2004). Furthermore, the brief interventions seem acceptable and feasible for school based practice as all teachers completed the intervention, the majority of the teachers reported to use the techniques learned at three months follow-up (Staff et al., 2021), and most of the teachers would recommend the training to colleagues. Such short individualized interventions well meet teachers’ needs (DuPaul et al., 2019; Egan et al., 2019; Gaastra et al., 2020), and fits with current ADHD guidelines suggesting that environmental modifications are regarded as first-line interventions prior to more intensified treatment (Akwa GGZ, 2019; National Institute for Health and Care Excellence, 2018). To increase suitability for schools, both sets of techniques could be combined into one intervention. For example, a brief and individualized intervention combing the effective sets of techniques can be provided to teachers seeking help to cope with the disruptive behavior of an individual student showing ADHD symptoms (e.g., Tier 2 interventions).

## Supplementary Information

Below is the link to the electronic supplementary material.

Supplementary file1 (DOCX 13 KB)

Supplementary file2 (DOCX 16 KB)

Supplementary file3 (DOCX 15 KB)

Supplementary file4 (DOCX 78 KB)

## Data Availability

The data that support the findings of this study are available from the corresponding author (AS), upon reasonable request.
