# Supplementary material for: Effectiveness of Specific Techniques in Behavioral Teacher Training for Childhood ADHD Behaviors: Secondary Analyses of a Randomized Controlled Microtrial
Source: Res Child Adolesc Psychopathol. 2022 Jan 11;50(7):867–80. doi: 10.1007/s10802-021-00892-z (PMC9246781; doi:10.1007/s10802-021-00892-z)
Supplement: Supplementary file 1 — Supplementary file1 (DOCX 13 KB) [file 10802_2021_892_MOESM1_ESM.docx]

| **Table A**. Description of the interventions provided in the antecedent and consequent condition. | | |
| --- | --- | --- |
| Session steps | *Specific to the antecedent condition* | *Specific to the consequent condition* |
| 1 Psycho-education on ADHD | Psycho-education on how stimuli evoke behaviors, executive functioning deficits in children with ADHD, and how antecedent-based techniques adapt to this by changing the discriminative value of stimuli | Psycho-education on how consequences affect behavior, altered reward sensitivity in children with ADHD, and how consequent-based techniques adapt to this by changing the consequences of behavior |
| 2 Selecting the problem behavior based on the frequency and severity of behavior |  |  |
| 3 Behavioral analysis | Identifying antecedents that elicited problem behavior | Identifying consequences that positively or negatively reinforce problem as well as desired behavior |
| 4 Defining desired target behaviors |  |  |
| 5 Intervention plan with behavioral techniques | Antecedent-based techniques were taught: setting clear rules, providing clear instructions, discussing challenging situations with the child in advance, and providing structure in time and space | Consequent-based techniques were taught: praise, reward, shaping, planned ignoring, and negative consequences |
| 6 Practicing the intervention plan |  |  |
| 7 Homework: implementing intervention plan for the coming week |  |  |

**Supplementary Material S1.**
