# Supplementary material for: Effectiveness of Specific Techniques in Behavioral Teacher Training for Childhood ADHD Behaviors: Secondary Analyses of a Randomized Controlled Microtrial
Source: Res Child Adolesc Psychopathol. 2022 Jan 11;50(7):867–80. doi: 10.1007/s10802-021-00892-z (PMC9246781; doi:10.1007/s10802-021-00892-z)
Supplement: Supplementary file 2 — Supplementary file2 (DOCX 16 KB) [file 10802_2021_892_MOESM2_ESM.docx]

**Supplementary Material S2.**

| **Table B**. Characteristics of the subset of the sample included in the analyses of classroom observations. | | | | |
| --- | --- | --- | --- | --- |
|  | AC (*n* = 20) | CC (*n* = 20) | WC (*n* = 20) | Group comparisons |
| Age at assessment in years | 8.60 (1.47) | 8.93 (1.45) | 8.27 (1.08) | *F*(2, 59) = 1.21, *p* = .306 |
| Sex, *n* (*%*) boys | 16 (80.0) | 15 (75.0) | 18 (90.0) | *χ*^2^ = 1.56, *p* = .459 |
| IQ | 99.30 (11.11) | 97.40 (12.09) | 103.85 (11.24) | *F*(2, 59) = 1.67, *p* = .198 |
| SES*^a^* | 5.28 (1.43) | 5.16 (1.12) | 4.90 (1.18) | *F*(2, 58) = .47, *p* = .627 |
| Caucasian, *n* (*%*) | 19 (95.0) | 17 (85.0) | 20 (100.0) | *Fisher’s exact =* 3.11, *p* = .310 |
| ADHD diagnosis, *n* (*%*) | 5 (25.0) | 4 (20.0) | 5 (25.0) | *χ*^2^ = .19, *p* = .911 |
| Other psychiatric diagnosis, *n* (*%*) | 0 (0) | 2 (10) | 0 (0) | *Fisher’s exact =* 2.77, *p* = .322 |
| TTI symptom severity  Inattention  Hyperactivity-Impulsivity  ODD  CD | 4.20 (1.64)  3.15 (1.79)  1.25 (1.48)  .00 (.00) | 5.00 (1.95)  4.65 (2.50)  .60 (.94)  .00 (.00) | 4.20 (1.47)  4.00 (1.69)  1.10 (1.41)  .00 (.00) | *F*(2, 59) = 1.48, *p* = .236  *F*(2, 59) = 2.77, *p* = .071  *F*(2, 59) = 1.37, *p* = .262 |
| DBDRS  Inattention  Hyperactivity-Impulsivity | 17.50 (5.61)  13.50 (6.44) | 17.60 (3.47)  15.25 (6.66) | 16.20 (5.44)  16.80 (6.83) | *F*(2, 59) = .50, *p* = .608  *F*(2, 59) = 1.24, *p* = .298 |
| IRS impairment,  Number of domains  Average score | 3.20 (.83)  6.51 (1.34) | 3.15 (1.18)  6.36 (1.76) | 3.06 (.93)  6.16 (1.35) | *F*(2, 55) = .09, *p* = .918  *F*(2, 55) = .25, *p* = .780 |
| *Teacher ratings* |  |  |  |  |
| SWAN  Inattention  Hyperactivity-Impulsivity | 15.50 (4.67)  14.05 (6.46) | 13.75 (5.19)  13.75 (5.97) | 15.05 (4.31)  14.95 (5.84) | *F*(2, 59) = .74, *p* = .484  *F*(2, 59) = .21, *p* = .811 |
| DBDRS  ODD | 9.10 (6.38) | 4.05 (3.58) | 8.95 (5.11) | *F*(2, 59) = 6.22, *p* = .004 (AC, WC > CC) |
| *Parent ratings^b^* |  |  |  |  |
| SWAN  Inattention  Hyperactivity-Impulsivity | 4.87 (9.09)  5.47 (7.91) | 9.16 (8.25)  9.63 (6.45) | 5.25 (4.52)  9.45 (6.60) | *F*(2, 53) = 1.89, *p* = .161  *F*(2, 53) = 1.87, *p* = .164 |
| DBDRS  ODD | 5.89 (4.11) | 5.37 (3.64) | 6.40 (4.63) | *F*(2, 54) = .30, *p* = .742 |
| *M* and *SD* are depicted unless otherwise stated.  *Note*. AC = antecedent condition; ADHD = attention-deficit/hyperactivity disorder; CC = consequent condition; CD = conduct disorder; DBDRS = Disruptive Behavior Disorder Rating Scale; IRS = Impairment Rating Scale; ODD = oppositional defiant disorder; SES = socioeconomic status; SWAN = Strengths’ and Weaknesses of ADHD and Normal Behavior; TTI = Teacher Telephone Interview; WC = waitlist control condition.  *^a^* SES was measured by parental educational level (average of both parents) through the Dutch classification system (1 = no education completed, 2 = early childhood education, 3 = primary education, 4 = lower secondary education, 5 = upper secondary education, 6 = undergraduate school, 7 = graduate school, 8 = post-graduate education) (CBS, 2016).  *^b^* missing parent ratings: 1 parent (CC) did not fill in any questionnaire, and 3 other parents (AC) did not fill in the SWAN. | | | | |
