# Supplementary material for: Effectiveness of Specific Techniques in Behavioral Teacher Training for Childhood ADHD Behaviors: Secondary Analyses of a Randomized Controlled Microtrial
Source: Res Child Adolesc Psychopathol. 2022 Jan 11;50(7):867–80. doi: 10.1007/s10802-021-00892-z (PMC9246781; doi:10.1007/s10802-021-00892-z)
Supplement: Supplementary file 3 — Supplementary file3 (DOCX 15 KB) [file 10802_2021_892_MOESM3_ESM.docx]

**Supplementary Material S3.**

| **Table C.** Means and standard deviations on all outcomes at all four time points. | | | | | | | | | | | | | |
| --- | --- | --- | --- | --- | --- | --- | --- | --- | --- | --- | --- | --- | --- |
|  |  | T0 |  |  | T1 |  |  | T2 |  |  | T3 |  |  |
|  |  | *N* | *M* | *SD* | *N* | *M* | *SD* | *N* | *M* | *SD* | *N* | *M* | *SD* |
| Teacher ratings |  |  |  |  |  |  |  |  |  |  |  |  |  |
| Inattention symptoms (SWAN) | AC | 30 | 15.03 | 4.41 | 27 | 11.19 | 6.38 | 29 | 13.17 | 5.92 | 26 | 9.38 | 6.04 |
|  | CC | 30 | 14.17 | 5.11 | 28 | 13.75 | 5.89 | 28 | 12.71 | 5.61 | 27 | 10.44 | 5.71 |
|  | WC | 30 | 15.07 | 5.07 | 27 | 15.74 | 5.97 | 25 | 15.92 | 4.85 |  |  |  |
| Hyperactivity-impulsivity symptoms (SWAN) | AC | 30 | 13.57 | 6.77 | 27 | 10.22 | 6.05 | 29 | 11.93 | 6.63 | 26 | 10.19 | 6.65 |
|  | CC | 30 | 13.77 | 6.35 | 28 | 12.93 | 7.29 | 28 | 12.32 | 6.79 | 27 | 10.04 | 7.18 |
|  | WC | 30 | 16.83 | 6.26 | 27 | 18.63 | 4.99 | 25 | 16.08 | 5.51 |  |  |  |
| ODD-symptoms (DBDRS) | AC | 30 | 8.00 | 6.45 | 27 | 6.85 | 5.55 | 29 | 6.62 | 5.62 | 26 | 5.92 | 6.17 |
|  | CC | 30 | 5.00 | 5.09 | 28 | 5.29 | 5.14 | 28 | 5.36 | 4.79 | 27 | 4.59 | 4.34 |
|  | WC | 30 | 8.97 | 5.32 | 27 | 9.00 | 5.74 | 25 | 8.28 | 5.81 |  |  |  |
| Impairment (IRS) average score | AC | 30 | 6.22 | 1.65 |  |  |  | 29 | 5.79 | 1.79 |  |  |  |
|  | CC | 30 | 6.14 | 1.97 |  |  |  | 28 | 5.77 | 1.90 |  |  |  |
|  | WC | 25 | 6.29 | 1.28 |  |  |  | 25 | 6.93 | 1.29 |  |  |  |
| Classroom observations*^a^* |  |  |  |  |  |  |  |  |  |  |  |  |  |
| Inattention (*%*) | AC | 20 | 27.23 | 15.96 |  |  |  | 18 | 26.05 | 14.65 |  |  |  |
|  | CC | 20 | 28.97 | 10.88 |  |  |  | 19 | 25.47 | 10.30 |  |  |  |
|  | WC | 20 | 30.56 | 16.66 |  |  |  | 20 | 36.33 | 19.93 |  |  |  |
| Motor hyperactivity (*%*) | AC | 20 | 30.37 | 19.63 |  |  |  | 18 | 27.13 | 16.90 |  |  |  |
|  | CC | 20 | 40.35 | 20.47 |  |  |  | 19 | 28.88 | 18.31 |  |  |  |
|  | WC | 20 | 32.60 | 15.67 |  |  |  | 20 | 33.13 | 18.20 |  |  |  |
| Verbal hyperactivity (*%*) | AC | 20 | 5.73 | 4.87 |  |  |  | 18 | 11.01 | 10.06 |  |  |  |
|  | CC | 20 | 9.08 | 7.83 |  |  |  | 19 | 6.22 | 3.83 |  |  |  |
|  | WC | 20 | 10.69 | 6.75 |  |  |  | 20 | 10.13 | 6.82 |  |  |  |
| Oppositional behavior (*K*) | AC | 20 | .30 | 1.13 |  |  |  | 18 | .10 | .31 |  |  |  |
|  | CC | 20 | .45 | 1.00 |  |  |  | 19 | .50 | 1.00 |  |  |  |
|  | WC | 20 | 1.65 | 3.08 |  |  |  | 20 | 1.75 | 3.34 |  |  |  |
| *Note*. AC = antecedent condition; ADHD = attention-deficit/hyperactivity disorder; CC = consequent condition; DBDRS = Disruptive Behavior Disorder Rating Scale; ODD = oppositional defiant disorder; IRS = Impairment Rating Scale; SWAN = Strengths’ and Weaknesses of ADHD and Normal behavior rating scale; WC = waitlist-control condition.  *^a^* Classroom observations were conducted in a subsample of children (*n* = 60). For descriptions of this sample see Supplementary Material S2 (Table B). | | | | | | | | | | | | | |
