# Supplementary material for: Effectiveness of Specific Techniques in Behavioral Teacher Training for Childhood ADHD Behaviors: Secondary Analyses of a Randomized Controlled Microtrial
Source: Res Child Adolesc Psychopathol. 2022 Jan 11;50(7):867–80. doi: 10.1007/s10802-021-00892-z (PMC9246781; doi:10.1007/s10802-021-00892-z)
Supplement: Supplementary file 4 — Supplementary file4 (DOCX 78 KB) [file 10802_2021_892_MOESM4_ESM.docx]

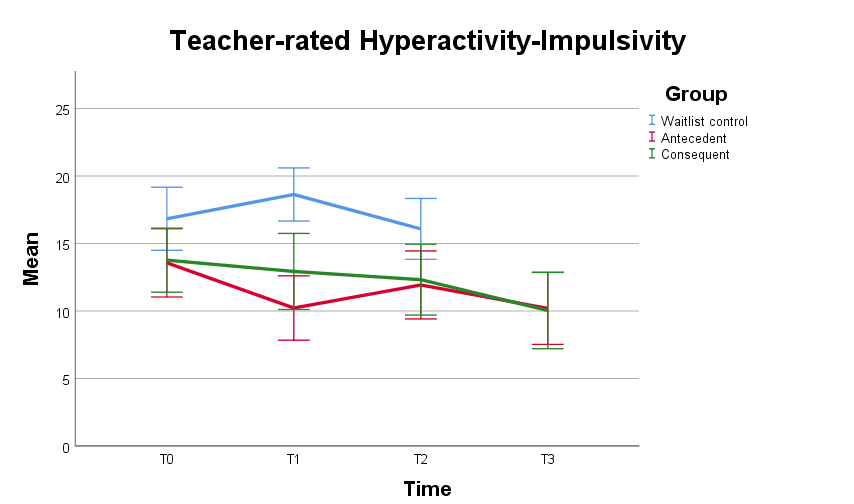
**Supplementary Material S4.**

**
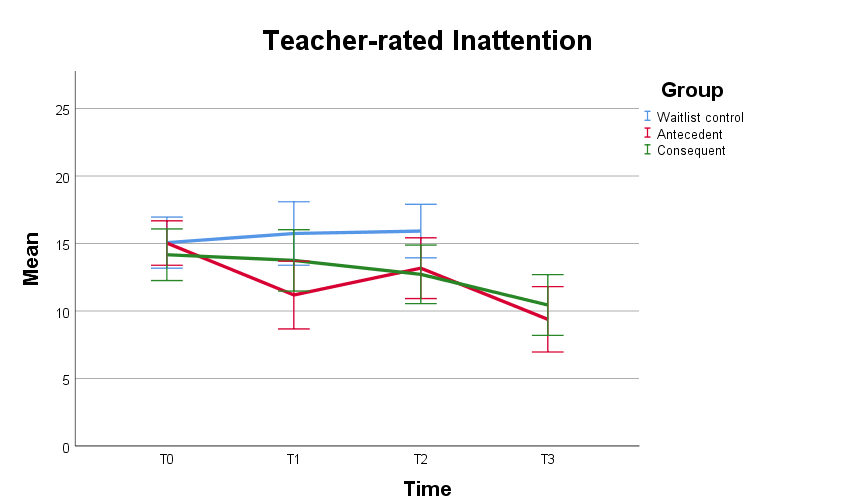
**


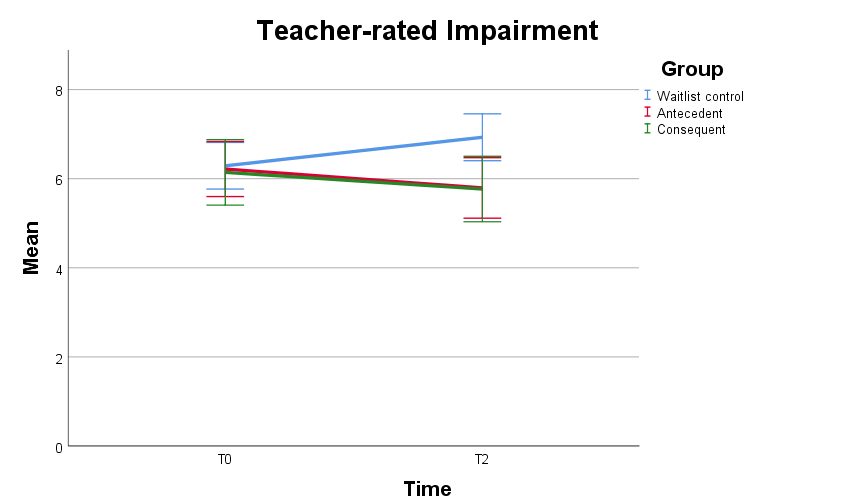

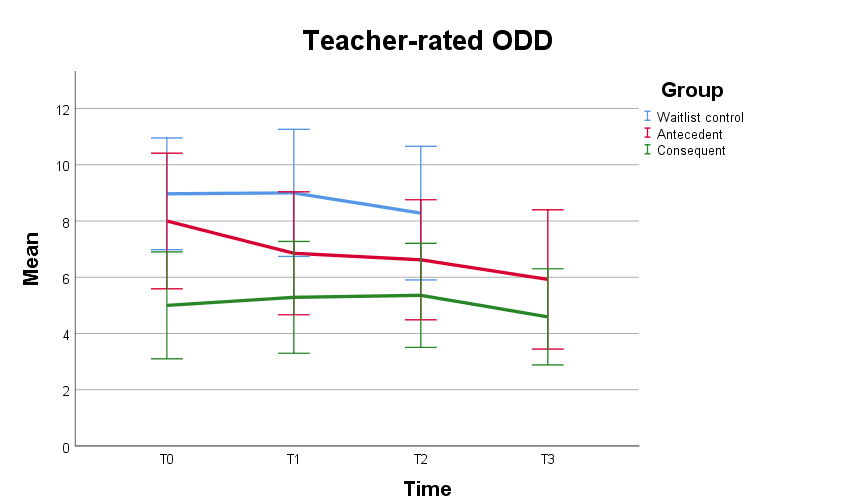


*Figure A.* Observed values for the development over time in the three conditions for all teacher-rated outcomes. Error bars represent 95% confidence intervals.
